# The Extent and Reasons for Dissatisfaction From Outpatients Provided With Pharmacy Services at Two Public Hospitals in Eastern Ethiopia

**DOI:** 10.3389/fphar.2018.01132

**Published:** 2018-10-12

**Authors:** Shambel Nigussie, Dumessa Edessa

**Affiliations:** Department of Clinical Pharmacy, School of Pharmacy, College of Health and Medical Sciences, Haramaya University, Harar, Ethiopia

**Keywords:** patient satisfaction, pharmacy services, dissatisfaction, associated factors, Ethiopia

## Abstract

**Background:** Satisfaction of patients for pharmaceutical services reflects their preferences and expectations, and the realities of care. It is critical to understand the extent of dissatisfaction for pharmaceutical services and its associated factors in order to optimize the required quality of the services provided. Therefore, this study is aimed to explore the extent and reasons for dissatisfaction from outpatients provided with the pharmacy services at Hiwot Fana Specialized University Hospital and Federal Harar Police Hospital in Harar, eastern Ethiopia.

**Methods:** An institution-based cross-sectional study was conducted on 844 outpatients. Data were collected by interviewer administered interviews that employed a structured questionnaire which was meant to estimate dissatisfaction/satisfaction of the outpatients for the pharmacy services provided using a 1–5 point LIKERT scale. SPSS version 20.0 was employed to analyze data. Accordingly, potential covariates were identified using chi-squared test and binary logistic regression analyses were undertaken to adjust for the covariates.

**Results:** The highest (61.1%) dissatisfaction was scored for lack of consistent availability of prescribed drug(s). Factors that showed significant association with dissatisfaction were marital divorce [adjusted odds ratio (AOR) 2.67; 95% CI 1.01–7.06]; lack of quality system or Auditable Pharmaceutical Transaction Services (AOR 13.56; 95% CI 9.10–20.23); and patients’ perceived insufficient knowledge of pharmacists (AOR 2.50; 95% CI 1.61–3.87) and good interaction with their pharmacists (AOR 0.28; 95% CI 0.14–0.56).

**Conclusion:** Outpatients’ highest dissatisfaction was related with the inadequate availability of prescribed drug(s). Lack of quality system; marital divorce; and patients’ perceived insufficient knowledge of pharmacists increased the likelihood of dissatisfaction but it was less likely to occur in outpatients who perceived their interaction with pharmacists as positive. Therefore, in addition to securing consistent availability of drugs and implementing a quality system, improving the technical and personal skills of pharmacists is likely to improve satisfaction of patients with the pharmacy services.

## Introduction

Sub-optimal pharmaceutical services may lead to inappropriate treatment with medications, prolongation or exacerbation of disease(s), and increment in cost of the treatment (Teshale et al., 2014). Patients who are satisfied with pharmaceutical services are more likely to take their medications appropriately and less likely to change from one healthcare to another (Briesacher and Corey, 1997). Hence, consistent provision of high-quality pharmaceutical services to patients that meets their expectation is a critical step in the process of maintaining such satisfaction (Correr et al., 2009). Satisfaction of patients for services can reflect their preferences and expectations, and the realities of care so that it can also serve as an essential tool to measure quality of pharmaceutical services (Briesacher and Corey, 1997). Therefore, understanding the extent of dissatisfaction with pharmaceutical services and its associated factors will be a key first step toward optimizing such aspects of healthcare (MacKinnon, 2005). In the provision of pharmaceutical services, a positive and meaningful patient–pharmacist communication is an opportunity through which pharmacists contribute to the efforts aimed to improve treatment outcomes of their patients (Erah and Chuks-Eboka, 2008).

Satisfaction of patients for pharmaceutical services is a multifactor concept which is very difficult to measure (Adwi et al., 2015). Various factors of patients such as income, perceived health, and insurance statuses are reported as predictors of the patients’ satisfaction for pharmacy services provided (Lee et al., 2015). Other factors indicated to influence the satisfaction of patients with the pharmacy services provided include accessibility, availability of prescribed drug(s), experience of patients for health facility visits, and attitude of pharmacy service providers (Eshetu and Gedif, 2010; Ahmad et al., 2016). To consistently optimize satisfaction of patients for pharmacy services, it is indispensable to identify factors which could strongly predict its aspect of dissatisfaction at different cohorts of population. Published data have described levels of patient satisfaction in terms of whether or not a given healthcare service is meeting the expectations and desires of patients it serves but not exhaustively identify key areas of quality improvement in the healthcare facility (Kalungia and Kamanga, 2016). Aspects such as persistent shortages or stock-outs of commonly prescribed drugs, long waiting times, and lack of privacy during medication counseling were highlighted as key areas for improvement of hospital pharmacy services (USAID/SIAPS, 2014; Kalungia and Kamanga, 2016).

In Ethiopia, few reports of previously published data described the extent and factors associated with the quality of health or pharmaceutical services (Oljira and Gebre-Selassie, 2001; Tateke et al., 2012; Surur et al., 2015; Woldeyohanes et al., 2015; Workye et al., 2015; Abdosh, 2016; Kefale et al., 2016; Berehe et al., 2018). To our knowledge, none of the published data have directly addressed the degree and reasons for dissatisfaction/satisfaction from outpatients with pharmacy services. In addition, generalization of few of the indicated factors of dissatisfaction from outpatients with the pharmacy services identified in other settings (countries) to the Ethiopian settings with variable cohorts of nations could be very difficult. Therefore, this study is aimed to explore the extent and reasons for dissatisfaction from outpatients provided with pharmaceutical services in two public hospitals of eastern Ethiopia.

## Materials and Methods

### Study Setting, Design, and Participants

The study was conducted in outpatient pharmacies of Hiwot Fana Specialized University Hospital (HFSUH) and Federal Harar Police Hospital (FHPH) in Harar, eastern Ethiopia, from February to June, 2016. An institution-based cross-sectional study was employed to investigate aspects contributing to dissatisfaction from outpatients provided with pharmaceutical services at the two public hospitals (HFSUH and FHPH). A total of 844 outpatients were enrolled for the study [422 patients from FHPH (a facility which has not yet started auditable pharmaceutical transaction services (APTS)) and 422 patients from HFSUH (a facility which has implemented APTS)]. The APTS is defined as a service delivery arrangement or system that aims to establish a transparent, auditable, and accountable medicines transaction and services at health facilities (FMOH, 2014). It is a quality system for outpatient pharmacy services and its implementation in hospitals is also well accepted by the Ethiopian Federal Ministry of Health in 2014.

Patients coming to either of the health facility during February to June, 2016 for pharmaceutical services were randomly selected and interviewed. The interview was made by using a structured questionnaire that involves satisfaction questions customized from the World Health Organization (WHO) criteria for satisfaction of pharmaceutical service provisions (FMHACA, 2012; MSH, 2012). A complete data for a questionnaire designed to assess patients’ perceived interaction status with pharmacists and other parameters aimed to assess factors influencing the exit knowledge of the patients for dispensed medicines was fully indicated in a previous publication that partly used the same dataset obtained from interview of patients served at HFSUH (Hirko and Edessa, 2017). However, data addressing satisfaction of patients with the pharmacy services at the two hospitals were not included in the previous publication.

Patients coming to the outpatient pharmacies of either HFSUH or HFPH and who were willing to be interviewed during the data collection period were enrolled for the study, while all severely sick patients were not considered. Sample size of the patients enrolled was calculated by the use of single population proportion formula. For the sample size calculation, it was considered to take α = 0.05 (two-tailed) and proportion of satisfaction for pharmaceutical services = 49.6% (*P* = 2.48 out of 5 on a LIKERT scale; Workye et al., 2015). Accordingly, after adding 10% of the calculated sample size for possible non-contingency, the final sample size was determined to be 422 for each study site which totals to 844 outpatients at the two study sites.

### Study Outcomes

Extent of dissatisfaction from outpatients provided with pharmaceutical services was the key primary outcome variable measured by the analysis. The dissatisfaction was defined by a score of ≤3 on 1–5 marks LIKERT scale for utmost three of the eight questions meant to measure satisfaction in the interview questionnaire. Accordingly, patient satisfaction refers to the extent to which an individual’s needs are met as defined by a score of >3 on LIKERT scale for at least five of the eight questions meant to measure satisfaction in the questionnaire. Median outpatients’ satisfaction score was also an outcome variable compared for the two study settings for all variables meant to measure the aspects of satisfaction.

### Data Quality and Collection Process

A structured interviewer administered questionnaire was employed for the data collection. The questionnaire had two parts. The first part specifically addressed socio-demographic characteristics of patients involving age, sex, educational levels, occupational status, marital status, and ethnicity. This part of the questionnaire also collected information of patient–pharmacist interaction aspects. The second part of the questionnaire focused on satisfaction of patients for quality of pharmacy services. To assure the data quality during the study, the structured questionnaire was extensively piloted before implementation and data collectors were trained to collect the data. Every day, after data collection, each datum was also reviewed and checked for completeness by the supervisor and the principal investigator, and the necessary feedback was given to the data collectors on the next day. Moreover, the study participants were also well oriented about the study topic focusing on its main objectives and advantages of answering the questions honestly.

### Statistical Analysis

Data were entered into an Epi-info version 3.5.1 and analyzed using SPSS version 20.0. Descriptive statistics were employed to summarize socio-demographic characteristics of the patients and their interaction status with pharmacists. Extent of dissatisfaction/satisfaction scores for the patients was ranked based on median and variance of the scores. A chi-squared (χ^2^) test was employed to identify potential covariates of dissatisfaction with pharmaceutical services. Binary logistic regression analyses were accomplished to determine associations between the different variables and the key outcome variable (dissatisfaction with pharmacy services). In all of the analyses, significance testing was done using two-sided *p*-values (*P*) and 95% confidence levels (95% CI). In all of such cases, *p*-value < 0.05 was considered significant.

## Results

Data were prospectively collected by interview of 844 outpatients (422 of them form HFSUH and 422 of them from FHPH). Characteristics of the patients interviewed at the two outpatient pharmacy settings are described in **Table 1**. Majority of the outpatients interviewed at HFSUH and FHPH, respectively, were male (59.2 and 50.9%), were in the age range of years 19–29 (36 and 31.5%), were married (60.9 and 70.6%), were at secondary (27.5 and 41.2%) and primary (26.8 and 28.7%) levels of education, were Oromo ethnic groups (56.2 and 41.9%), and were urban residents (63 and 83.9%). In addition, majority of the patients interviewed at HFSUH and FHPH, respectively, reported their positive interaction with pharmacists in the outpatient pharmacy unit (64 and 68.2%); and also indicated clear voice and tone (83.4 and 87.2%), positive politeness (84.1 and 82%), clear instruction (81 and 79.4%), and sufficient knowledge of the pharmacists (64.9 and 64.7%).

**Table 1:** Socio-demographic characteristics and interaction of outpatients with pharmacy unit at HFSUH and HFPH, Harar, February–June, 2016.

Characteristics	Category	
	HFSUH-number (%)	HFPH-number (%)
**Gender**		
Male	250 (59.2)	215 (50.9)
Female	172 (40.8)	207 (49.1)
**Age (years)**		
≤18	28 (6.6)	13 (3.1)
19–29	152 (36)	133 (31.5)
30–39	123 (29.1)	111 (26.3)
40–49	73 (17.3)	81 (19.2)
50–59	34 (8.1)	45 (10.7)
≥60	12 (2.9)	39 (9.2)
**Marital status**		
Single/under age	136 (32.2)	90 (21.3)
Married	257 (60.9)	298 (70.6)
Divorced	17 (4)	13 (3.1)
Widowed	8 (1.9)	19 (4.5)
Separated	4 (1.0)	2 (0.5)
**Educational status**		
Illiterate	70 (16.6)	25 (5.9)
Read and write	30 (7.1)	32 (7.6)
Primary	113 (26.8)	121 (28.7)
Secondary	116 (27.5)	174 (41.2)
Tertiary	93 (22)	70 (16.6)
**Occupation**		
Farmer	103 (24.4)	5 (1.2)
Government employee	124 (29.3)	200 (47.4)
Merchant	58 (13.7)	56 (13.3)
Laborer	19 (4.5)	14 (3.3)
Private employee	19 (4.5)	28 (6.6)
Student	79 (18.9)	43 (10.2)
Housewife	17 (4)	48 (11.4)
Retirement	3 (0.7)	28 (6.6)
**Religion**		
Muslim	233 (55.2)	148 (35.1)
Orthodox	130 (30.8)	205 (48.6)
Protestant	59 (14)	69 (16.3)
**Ethnicity**		
Oromo	237 (56.1)	177 (41.9)
Harari	34 (8.1)	44 (10.4)
Amhara	99 (23.5)	157 (37.2)
Tigrie	25 (5.9)	27 (6.4)
Somali	19 (4.5)	10 (2.4)
Other^∗^	8 (1.9)	7 (1.7)
**Residence**		
Urban	266 (63)	354 (83.9)
Rural	156 (37)	68 (16.1)
**Pharmacy visit frequency**		
First time	70 (16.6)	24 (5.7)
Second time	130 (30.8)	85 (20.1)
Repeated times	222 (52.6)	313 (74.2)
**Income per month (Birr)**		
<724	151 (40.5)	120 (28.4)
≥724	251 (59.5)	302 (71.6)
**Perceived interaction status with pharmacist rated by patient**		
Poor	31 (7.3)	30 (7.1)
Fair	121 (28.7)	104 (24.7)
Good	270 (64)	288 (68.2)
**Primary language communicated by patient**		
Amharic	207 (49.0)	278 (65.9)
Afan Oromo	185 (43.8)	113 (26.8)
Adarigna	15 (3.6)	19 (4.5)
Somali	15 (3.6)	5 (1.2)
Tigrigna		7 (1.6)
**Perceived clearness of pharmacist’s voice and tone**		
Clear	352 (83.4)	368 (87.2)
Not clear	70 (16.6)	54 (12.8)
**Perceived comfort of waiting area**		
Not comfortable	41 (9.7)	73 (17.3)
Fairly comfortable	60 (14.2)	72 (17.1)
Comfortable	321 (76.1)	277 (65.6)
**Perceived politeness of service provider**		
Polite	355 (84.1)	346 (82)
Fairly polite	42 (10)	49 (11.6)
impolite	25 (5.9)	27 (6.4)
**Perceived clearness of the pharmacist’s instruction for patient**		
Clear	342 (81)	335 (79.4)
Fairly clear	43 (10.2)	48 (11.4)
Not clear	37 (8.8)	39 (9.2)
**Perceived sufficiency of pharmacist’s knowledge**		
Enough	274 (64.9)	273 (64.7)
Not enough	99 (23.5)	115 (27.2)
Don’t know	49 (11.6)	34 (8.1)

*Other^∗^ stands for Gurage, Argoba, and other minor ethnic groups in the area*.

This study also investigated the extent of dissatisfaction/satisfaction for pharmacy services (**Table 2** and **Supplementary Table S1**). Accordingly, the extent of outpatients’ satisfaction for the pharmacy services was scored by the use of 1–5 point on LIKERT scale. As a result, 32.2, 21.0, and 19.7% of the outpatients were very satisfied with the respect the pharmacists gave to them, with duration of waiting time for service, and with the time given for filling prescription, respectively. However, certain proportion of the patients was very dissatisfied with the availability of prescribed drugs (11.4%) and privacy in dispensing area (4.7%).

**Table 2:** Extent of patients’ satisfaction with the pharmacy services given at the outpatient pharmacies of HFSUH and FHPH, Harar, February–June, 2016.

Variable	Satisfaction level—number (%)				
	5	4	3	2	1
Suitability of dispensing area	118 (14.0)	409 (48.5)	234 (27.7)	59 (7.0)	24 (2.8)
Privacy in dispensing area	115 (13.6)	233 (27.6)	203 (24.1)	253 (30)	40 (4.7)
Degree of enjoyment with pharmacy staff’s service	115 (13.6)	428 (50.7)	211 (25.0)	70 (8.3)	20 (2.4)
Waiting time for service	177 (21.0)	452 (53.6)	136 (16.1)	61 (7.2)	18 (2.1)
Availability of prescribed drug(s)	81 (9.60)	247 (29.3)	219 (25.9)	201 (23.8)	96 (11.4)
Respect of pharmacist to patient	272 (32.2)	287 (34.0)	218 (25.8)	58 (6.9)	9 (1.10)
Relative location of outpatient pharmacy	89 (10.5)	435 (51.5)	229 (27.1)	78 (9.2)	13 (1.5)
Time given for filling prescription	166 (19.7)	436 (51.7)	191 (22.6)	41 (4.9)	10 (1.2)

*Note: 1—very dissatisfied, 2—dissatisfied, 3—neutral, 4—satisfied, and 5—very satisfied*.

Median satisfaction score investigated on the 1–5 point LIKERT scale was also rated for the pharmacy services given at the outpatient pharmacies of HFSUH and FHPH (**Table 3**). As indicated in the table, respect of pharmacists for patients (5.0 ± 0.60 for HFSUH and 4.0 ± 0.62 for FHPH) and the waiting time for service reception (4.0 ± 0.55 for HFSUH and 4.0 ± 0.92 for FHPH) were ranked as the first and second highest satisfaction scores at the two hospitals.

**Table 3:** Median satisfaction score ranked for the pharmacy services given at outpatient pharmacies of HFSUH and FHPH, Harar, February–June, 2016.

Variable of satisfaction	Median of satisfaction score ± variance		
	HFSUH	FHPH	Overall
Respect of pharmacist to patient	5.0 ± 0.60	4.0 ± 0.62	4.0 ± 0.94
Waiting time for service	4.0 ± 0.55	4.0 ± 0.92	4.0 ± 0.83
Waiting time for filling prescription	4.0 ± 0.70	4.0 ± 0.90	4.0 ± 0.70
Enjoyment for pharmacy staff’s service	4.0 ± 0.52	4.0 ± 1.01	4.0 ± 0.80
Suitability of dispensing area and encounter	4.0 ± 0.50	3.0 ± 0.96	4.0 ± 0.82
Location of outpatient pharmacy relative to other service areas	4.0 ± 0.55	3.0 ± 0.78	4.0 ± 0.73
Privacy in dispensing area	4.0 ± 0.76	2.0 ± 1.15	3.0 ± 1.28
Availability of prescribed drug(s)	4.0 ± 0.85	2.0 ± 1.33	3.0 ± 1.37

Following identification of potential covariates, series of binary logistic regression analyses were accomplished in order to assess factors associated with dissatisfaction for the pharmacy services at the two outpatient pharmacies. Accordingly, a final multivariable regression analysis conducted to adjust for the potential covariates indicated associations of few variables with the patients’ dissatisfaction for pharmacy services at the two hospitals. To this end, a significantly increased likelihood of dissatisfaction for outpatient pharmacy services had occurred among patients with marital divorce [adjusted odds ratio (AOR) 2.67; 95% CI 1.01–7.06], who were served in a setting which lacks a quality system (APTS; AOR 13.56; 95% CI 9.10–20.23); and who perceived knowledge of pharmacists as insufficient (AOR 2.50; 95% CI 1.61–3.87) compared to their respective reference categories. However, the dissatisfaction for pharmacy services in the two outpatient pharmacies was less likely to occur among the patients who perceived fair (AOR 0.46; 95% CI 0.23–0.93) or good (AOR 0.28; 95% CI 0.14–0.56) interactions with pharmacists and who were communicating in Afan Oromo as their primary language with pharmacists (AOR 0.54; 95% CI 0.33–0.88) compared to their respective reference categories (**Table 4**).

**Table 4:** Regression analysis for factors associated with dissatisfaction of outpatients for pharmacy services at HFSUH and FHPH, Harar, February–June, 2016.

Variable	COR [95% CI]	*P*-values	AOR [95% CI]	*P*-values
**Age (year)**				
≤18	1		1	
19–29	1.61 [0.77–3.35]	0.20	1.64 [0.64–4.24]	0.30
30–39	1.47 [0.70–3.08]	0.30	2.0 [0.73–5.48]	0.17
40–49	1.69 [0.79–3.63]	0.17	1.85 [0.66–5.18]	0.24
50–59	1.33 [0.58–3.08]	0.49	0.92 [0.30–2.80]	0.88
≥60	3.89 [1.60–9.46]	0.003	1.99 [0.62–6.38]	0.24
**Religion**				
Muslim	1		1	
Orthodox	1.35 [0.99–1.83]	0.054	0.76 [0.49–1. 16]	0.20
Protestant	1.34 [0.88–2.03]	0.16	0.65 [0.37–1.14]	0.14
**Marital status**				
Single or under age	1		1	
Married	1.26 [0.91–1.75]	0.158	1.21 [0.75–1.97]	0.43
Divorced	2.09 [0.97–4.52]	0.059	2.67 [1.01–7.06]	0.048^∗^
Widowed	2.62 [1.16–5.88]	0.02	2.14 [0.73–6.24]	0.17
Separated	1.05 [0.19–5.85]	0.97	1.33 [0.12–14.66]	0.81
**Setting of health facility**				
HFSUH (implemented APTS)	1		1	
FHPH (not implemented APTS)	11.28[7.96–16.0]	<0.001	13.56[9.10–20.23]	<0.001^∗^
**Perceived patient–pharmacist interaction status**				
Poor	1		1	
Fair	0.50 [0.28–0.90]	0.02	0.46 [0.23–0.93]	0.031^∗^
Good	0.34 [0.19–0.58]	<0.001	0.28 [0.14–0.56]	<0.001^∗^
**Primary language communicated by patient**				
Amharic	1		1	
Afan Oromo	0.50 [0.36–0.68]	<0.001	0.54 [0.33–0.88]	0.015^∗^
Adarigna	0.95 0.47–1.94]	0.90	0.68 [0.27–1.71]	0.41
Somali	1.36 [0.55–3.34]	0.50	3.03 [0.89–10.35]	0.07
Tigrigna	3.41 [0.65–17.8]	0.14	1.25 [0.21–7.41]	0.80
**Perceived clearness of pharmacist’s voice and tone**				
Clear	1		1	
Not clear	1.36 [0.93–2.00]	0.114	1.03 [0.60–1.77]	0.90
**Perceived comfort of waiting area**				
Not comfortable	1		1	
Fairly comfortable	0.78 [0.47–1.29]	0.35	1.04 [0.57–1.92]	0.88
Comfortable	0.36 [0.24–0.54]	<0.001	0.67 [0.40–1.13]	0.13
**Perceived politeness of service provider**				
Polite	1		1	
Fairly polite	1.78 [1.15–2.77]	0.01	1.05 [0.58–1.90]	0.88
Impolite	2.40 [1.36–4.25]	0.002	1.41 [0.64–3.14]	0.39
**Perceived clearness of pharmacist’s instruction for patient**				
Clear	1		1	
Fairly clear	2.01 [1.29–3.13]	0.002	1.40 [0.77–2.55]	0.27
Not clear	2.19 [1.36–3.53]	0.001	1.62 [0.82–3.24]	0.16
**Perceived knowledge status of pharmacist**				
Sufficient	1		1	
Not sufficient	2.67 [1.93–3.69]	<0.001	2.50 [1.61–3.87]	<0.001^∗^
Don’t know	1.27 [0.79–2.07]	0.32	1.11 [0.59–2.12]	0.74

*Asterisk (^∗^) shows significant difference; COR, crude odds ratio; AOR, adjusted odds ratio*.

## Discussion

The present study indicated that the highest satisfaction score of outpatients with regard to quality of pharmaceutical services at HFSUH and FHPH was for respect of pharmacists to patients but their ranking for availability of prescribed drug(s) was least scored. Dissatisfaction for pharmaceutical services in the study settings was more likely to increase among outpatients with marital divorce, who were served in FHPH, and who perceived knowledge of their pharmacists as insufficient. Conversely, the dissatisfaction was less likely to occur among patients who perceived their interaction with pharmacists as fair or good and who primarily communicate in Afan Oromo. Therefore, marital divorce, insufficient knowledge of pharmacist, quality system (APTS) implementation status by the health facilities, status of patient–pharmacist interaction, and primary language of communication during the services provision were the factors associated with dissatisfaction for pharmaceutical services at the outpatient pharmacies of the study settings and all of them are discussed separately.

Lack of APTS implementation as a quality system in FHPH was indicated to have a 13.56 times higher odds of dissatisfaction from outpatients with pharmacy services. In agreement with this finding, published data confirmed that the APTS can improve patient satisfaction for pharmacy services (FMOH, 2014) and the overall performance of the pharmacy services (USAID/SIAPS, 2014; Adinew et al., 2015). Dissatisfaction from outpatients with pharmacy services was also 2.67 times more likely among outpatients with marital divorce compared to the patients who were single or under age. Similarly, published data reported that marital divorce affected an adult’s life-satisfaction or happiness greatly and it is also a period of tension, instability, isolation, hurt feelings, and often unfriendliness; all of which could increase the likelihood of dissatisfaction and missed medication doses (Ambert, 2009; Reis and Sprecher, 2009; Huang et al., 2013).

Moreover, dissatisfaction from outpatients with pharmacy services was 2.50 times more likely among patients who perceived knowledge of pharmacists as insufficient compared to the patients who perceived the knowledge as sufficient. Consistently, published data described that presence of knowledgeable pharmacists with regard to drugs, their side effects, and interaction or incompatibilities was a main reason and influential factor for patients to visit the pharmacy (Perepelkin, 2011; Khdour and Hallak, 2012). Moreover, perception of outpatients about the technical knowledge of pharmacists was also indicated to independently affect their satisfaction and quality of the patient–pharmacist interactions (Alghurair et al., 2012). Patients who perceived instruction and politeness of pharmacists as satisfactory and positive, respectively, were identified to have good knowledge for dispensed medications at exit from outpatient pharmacy of HFSUH (Hirko and Edessa, 2017). This study had investigated perception of outpatients pertaining to knowledge of pharmacists about medications they are dispensing and their communication; however, it did not measure ethnicity and language of preference for the pharmacists.

By this study, outpatients who perceived their interaction with pharmacists as fair and good had 0.46 and 0.28 times decreased likelihoods of dissatisfaction for pharmacy services, respectively. Similarly, a systematic review of published data reported that cooperation of patients can be hindered or enhanced by the behavior of health care professionals (Stevenson et al., 2004). It was also indicated that interaction quality between patients and pharmacists appeared to influence patient satisfaction and relationship obligation (Alghurair et al., 2012). This interaction quality can also be affected by the language of communication. Accordingly, outpatients who were communicating in Afan Oromo as their primary language had 0.54 times less likelihood of dissatisfaction from outpatients with pharmacy services. Several published data also confirmed the need of addressing communication barriers as a vital mechanism for pharmacists to be able to provide a pharmaceutical care that is aligned with the culture of patients (Mullins et al., 2005; Haack, 2008; Hulten et al., 2011). Similarly, language skills of pharmacists influenced their ability to counsel patients (Jacobs et al., 2006; Dilworth et al., 2009; Hussainy et al., 2012) so that their interaction through communication could affect satisfaction or dissatisfaction of the patients with the services they provide. Inadequate communication due to language difficulty from either side can have an adverse consequence that can demotivate patients to seek their usual source of pharmaceutical care (Teshale et al., 2014).

### Strength and Limitation of the Study

The study was designed to prospectively investigate the extent and reasons for dissatisfaction from outpatients with pharmacy services. Finding of the study is of importance to improving the quality of the pharmaceutical services provision in the study facilities and elsewhere with a range of different populations with regard to a language, ethnicity, and cultural perspectives. Though study concept, design, and statistical tools used were appropriate for reliable outcome, there were some limitations to this study. First, no research hypotheses were made prior to the study and precision for strong prediction of the few factors identified could be limited due to their wide confidence interval. Second, pharmaceutical service providers at the two different hospitals selected for the study are different in terms of their language, technical, and personal skills. However, since the study design was to identify perspectives of patients with regard to quality of the pharmaceutical services provided, language, ethnicity, and other aspects of the pharmacy service providers were not investigated. These limitations could result into under- or over-estimations of the dissatisfaction from outpatients with the pharmacy services. Therefore, any interpretation of the findings in this study needs to be made in consideration of aforementioned limitations.

## Conclusion

The present study clearly described the extent and reasons for dissatisfaction from outpatients provided with pharmacy services. The respect of pharmacists toward their patients and availability of prescribed drug(s) were aspects with the highest and least scored satisfactions by the outpatients from the study settings, respectively. Marital divorce, lack of quality system, and perceived insufficient knowledge of pharmacists were the predictors of dissatisfaction from outpatients provided with the pharmacy services identified by this study. However, pharmaceutical services provided by pharmacists through counseling in primary language of the patients supplemented with positive patient–pharmacist interaction were indicated to lessen the likelihoods of dissatisfaction. Therefore, in addition to securing consistent availability of prescribed drug(s) and implementing a quality system, improving the technical and personal skills of pharmacists is likely to increase satisfaction for pharmacy services at outpatients and improve health care.

## Data Availability

All relevant data are included in the manuscript and supporting information files.

## Ethics Statement

The study was approved by Haramaya University, College of Health and Medical Sciences, School of Pharmacy (SOP/874/02/2016). Permission to interview the study participants was obtained officially from the management offices of the two hospitals from where the study participants were selected. Informed consent was obtained from each participant prior to the interview and confidentiality of the information was also ensured.

## Author Contributions

SN carried out the overall design, execution of the study, took charge of data collection, and participated in drafting of the manuscript. DE also conceived the study, carried out the overall design and execution of the study, took charge of data collection, and performed statistical analysis and drafting of the manuscript. Both of the authors read and approved the manuscript.

## Conflict of Interest Statement

The authors declare that the research was conducted in the absence of any commercial or financial relationships that could be construed as a potential conflict of interest.

## References

[B1] Abdosh B. (2016). The quality of hospital services in eastern Ethiopia: patient’s perspective. *Ethiop. J. Health Dev.* 20 199–200.

[B2] Adinew A. Ololo S. Tessema F. (2015). *Evaluation of the Implementation Status, Outcomes and Challenges of “Auditable Pharmaceuticals Transactions and Services” in Ten Selected Hospitals of Ethiopia.* Master’s thesis, Jimma University, Addis Ababa, 1–79.

[B3] Adwi G. M. E. Almahdi A. F. Elkhawad A. O. (2015). An investigation of patient satisfaction with pharmaceutical care services. *World J. Pharm. Pharm. Sci.* 4 180–191.

[B4] Ahmad A. M. K. Alghamdi M. A. S. Alghamdi S. A. S. Alsharqi A. Z. Al-Borie H. M. (2016). Factors influencing patient satisfaction with pharmacy services: an empirical investigation at king fahd armed forces hospital, Saudi Arabia. *Int. J. Bus. Manag.* 11 272–280. 10.5539/ijbm.v11n9p272

[B5] Alghurair S. A. Simpson S. H. Guirguis L. M. (2012). What elements of the patient–pharmacist relationship are associated with patient satisfaction? *Patient Prefer. Adherence* 6 663–676. 10.2147/PPA.S35688 23055699 PMC3461603

[B6] Ambert A. M. (2009). *Divorce: Facts, Causes & Consequences*, 3rd Edn. Ottawa, ON: The Vanier Institute of The Family, 1–33.

[B7] Berehe T. T. Bekele G. E. Yimer Y. S. Lozza T. Z. (2018). Assessment of clients satisfaction with outpatient services at Yekatit 12 hospital medical college, Addis Ababa, Ethiopia. *BMC Res. Notes* 11:507. 10.1186/s13104-018-3603-3 30053830 PMC6063000

[B8] Briesacher B. Corey R. (1997). Patient satisfaction with pharmaceutical services at independent and chain pharmacies. *Am. J. Health Syst. Pharm.* 54 531–536. 9066860 10.1093/ajhp/54.5.531

[B9] Correr C. J. Pontarolo R. Souza R. A. P. Venson R. Melchiors A. C. Wiens A. (2009). Effect of a pharmaceutical care program on quality of life and satisfaction with pharmacy services in patients with type 2 diabetes mellitus. *Braz. J. Pharm. Sci.* 45 810–817. 10.1590/S1984-82502009000400027

[B10] Dilworth T. Mott D. Young H. (2009). Pharmacists’ communication with Spanish-speaking patients: a review of the literature to establish an agenda for future research. *Res. Soc. Adm. Pharm.* 5 108–120. 10.1016/j.sapharm.2008.05.005 19524859 PMC2875142

[B11] Erah P. O. Chuks-Eboka N. A. (2008). Patients’ perception of the benefits of pharmaceutical care services in the management of hypertension in a tertiary health care facility in benin city. *Trop. J. Pharm. Res.* 7 897–905. 10.4314/tjpr.v7i1.14674

[B12] Eshetu E. Gedif T. (2010). *Quality of Pharmaceutical Care in Government Hospitals of Addis Ababa.* Ph.D. thesis, Addis Ababa University, Addis Ababa, 1–68.

[B13] FMHACA (2012). *Manual for Medicines Good Dispensing Practice*, 2nd Edn. Addis Ababa: FMHACA, 1–76.

[B14] FMOH (2014). “Auditable pharmaceutical transaction and service (APTS) scaling up and making it functional,” in *Status Ways Forward. Federal Ministry of Health 16th National Annual 2014. Review Meeting Group Discussion*, (Abuja: Federal Ministry of Health).

[B15] Haack S. (2008). Engaging pharmacy students with diverse patient populations to improve cultural competence. *Am. J. Pharm. Edu.* 72:124. 19214278 10.5688/aj7205124 PMC2630151

[B16] Hirko N. Edessa D. (2017). Factors influencing the exit knowledge of patients for dispensed drugs at outpatient pharmacy of Hiwot Fana specialized university hospital, Eastern Ethiopia. *Patient Prefer. Adherence* 11 205–212. 10.2147/PPA.S128658 28223781 PMC5308573

[B17] Huang H. L. Li Y. C. J. Chou Y. C. Hsieh Y. W. Kuo F. Tsai W. C. (2013). Effects of and satisfaction with short message service reminders for patient medication adherence: a randomized controlled study. *BMC Med. Inform. Decis. Mak.* 13:127. 10.1186/1472-6947-13-127 24238397 PMC4225681

[B18] Hulten R. V. Blom L. Mattheusens J. Wolters M. Bouvy M. (2011). Communication with patients who are dispensed a first prescription of chronic medication in the community pharmacy. *Patient Edu. Couns.* 83 417–422. 10.1016/j.pec.2011.05.020 21621948

[B19] Hussainy S. Y. Styles K. Duncan G. (2012). Instructional design and assessment: a virtual practice environment to develop communication skills in pharmacy students. *Am. J. Pharm. Edu.* 76 202. 10.5688/ajpe7610202 23275667 PMC3530064

[B20] Jacobs E. Chen A. H. Karliner L. S. Agger-Gupt A. N. Mutha S. (2006). The need for more research on language barriers in health care: a proposed research Agenda. *Blackwell Publish.* 84 111–133. 16529570 10.1111/j.1468-0009.2006.00440.x PMC2690153

[B21] Kalungia A. C. Kamanga T. (2016). Patients’ satisfaction with outpatient pharmacy services at the university teaching hospital and Ndola central hospital in Zambia. *J. Prev. Rehabil. Med.* 1 13–16. 10.21617/jprm.2016.0101.3

[B22] Kefale A. T. Atsebah G. H. Mega T. A. (2016). Clients’ perception and satisfaction toward service provided by pharmacy professionals at a teaching hospital in Ethiopia. *Integr. Pharm. Res. Prac.* 5:85. 10.2147/IPRP.S118657 29354544 PMC5741042

[B23] Khdour M. R. Hallak H. O. (2012). Societal perspectives on community pharmacy services in West Bank - Palestine. *Pharm. Prac.* 10 17–24. 24155812 10.4321/s1886-36552012000100004 PMC3798164

[B24] Lee S. Godwin O. P. Kim K. Lee E. (2015). Predictive factors of patient satisfaction with pharmacy services in south korea: a cross-sectional study of national level data. *PLoS One* 10:e0142269. 10.1371/journal.pone.0142269 26540165 PMC4634764

[B25] MacKinnon K. J. (2005). *Striving Beyond Patient Satisfaction: A Roadmap for Pharmacists.* Bartlett, IL: Internet Continuing Education (InetCE).

[B26] MSH (2012). “Ensuring good dispensing practices,” in *MDS3 – Managing Access to Medicines and Health Technologies*, eds Spivey P., Consultant U. K. Arlington, VA: Management Science for Health.

[B27] Mullins C. D. Blatt L. Gbarayor C. M. Yang H. W. K. Baquet C. (2005). Health disparities: a barrier to high-quality care. *Am. J. Health Syst. Pharm.* 62 1873–1882. 10.2146/ajhp050064 16141106 PMC3262677

[B28] Oljira L. Gebre-Selassie S. (2001). Satisfaction with outpatient health services at Jimma hospital, South West Ethiopia. *Ethiop. J. Health Dev.* 15 179–184.

[B29] Perepelkin J. (2011). Public opinion of pharmacists and pharmacist prescribing. *Can. Pharm. J.* 144 86–93. 10.3821/1913-701X-144.2.86

[B30] Reis H. T. Sprecher S. (2009). *Divorce, Effects on Adults.* Thousand Oaks, CA: SAGE, 459–461.

[B31] Stevenson F. A. Cox K. Britten N. Dundar Y. (2004). A systematic review of the research on communication between patients and health care professionals about medicines: the consequences for concordance. *Health Expect.* 7 235–245. 10.1111/j.1369-7625.2004.00281.x 15327462 PMC5060245

[B32] Surur A. S. Teni F. S. Girmay G. Moges E. Tesfa M. Abraha M. (2015). Satisfaction of clients with the services of an outpatient pharmacy at a university hospital in northwestern Ethiopia: a cross-sectional study. *BMC Health Serv. Res.* 15:229. 10.1186/s12913-015-0900-6 26062912 PMC4464722

[B33] Tateke T. Woldie M. Ololo S. (2012). Determinants of patient satisfaction with outpatient health services at public and private hospitals in Addis Ababa, Ethiopia. *Afr. J. Prim. Health Care Fam. Med.* 4:a384 10.4102/phcfm.v4i1.384

[B34] Teshale C. Hussein J. Mussa S. (2014). Assessment of the quality of pharmaceutical service in Jimma Zone, Oromia regional state, South West Ethiopia. *Int. J. Pharm. Teach. Prac.* 5 60–66.

[B35] USAID/SIAPS (2014). *Auditable Pharmaceutical Transactions and Services (APTS): Findings of the Baseline Assessment at Federal, Addis Ababa, and Teaching Hospitals.* Arlington, VA: Management Sciences for Health, 1–27.

[B36] Woldeyohanes T. R. Woldehaimanot T. E. Kerie M. W. Mengistie M. A. Yesuf E. A. (2015). Perceived patient satisfaction with in-patient services at Jimma University specialized hospital, Southwest Ethiopia. *BMC Res. Notes* 8:285. 10.1186/s13104-015-1179-8 26126658 PMC4487793

[B37] Workye M. Admasu S. Abura T. Belete Y. Getaye Y. Teni F. S. (2015). Clients’ expectations from and satisfaction with medicine retail outlets in Gondar town, northwestern Ethiopia: a cross-sectional study. *Integr. Pharm. Res. Prac.* 4 1–12. 10.2147/IPRP.S75819 29354515 PMC5741013

