# Supplementary material for: The Extent and Reasons for Dissatisfaction From Outpatients Provided With Pharmacy Services at Two Public Hospitals in Eastern Ethiopia
Source: Front Pharmacol. 2018 Oct 12;9:1132. doi: 10.3389/fphar.2018.01132 (PMC6194162; doi:10.3389/fphar.2018.01132)
Supplement: Supplementary file 1 [file Data_Sheet_1.PDF]

Re-categorizing satisfactions marked as “5” and “4” as “satisfied” and those marked as “3”, “2” and “1” as “dissatisfaction”, levels of dissatisfaction for pharmacy services at outpatient pharmacies with regard to drug availability, privacy in dispensing area, location of outpatient pharmacy relative to other service areas was 61.1%, 58.8% and 48.6%, respectively, at the study sites (Table S1). However, dissatisfaction for respect of pharmacist for clients (15.1%) was the least level scored by patients interviewed.

Table S1: Levels of dissatisfaction from outpatients with pharmaceutical services at HFSUH and FHPH, Harar, June 2016

Variable	Satisfaction status	
	**Dissatisfied—No (%)**	**Satisfied—No (%)**
Satisfaction status with respect of pharmacy service provider for patient	127 (15.1)	717 (84.9)
Satisfaction status for waiting time for service	215 (25.5)	629 (74.5)
Satisfaction status for waiting time of filling prescription	218 (25.8)	626 (74.2)
Satisfaction status for pharmacy staff’s service	301 (35.7)	543 (64.3)
Satisfaction status for suitability of dispensing area and encounters	317 (37.6)	527 (62.4)

Satisfaction status for location of outpatient pharmacy relative to other service areas	410 (48.6)	434 (51.4)
Satisfaction status for privacy in dispensing area	496 (58.8)	348 (41.2)
Satisfaction status for continuous availability of prescribed medication (s)	516 (61.1)	328 (38.9)
